# The Treatment of Pneumonia in Children

**Published:** 1899-02

**Authors:** 


### THE TREATMENT OF PNEUMONIA IN CHILDREN.

The symposium convened by the chairman of the pediatric section has done practitioners a great service, in that it has presented the modern treatment of this disease so clearly that there can be no misconception. There existed a consensus of opinion upon the importance of absolute quiet, best obtained by complete isolation from other children; upon the need of careful nursing and abstaining from meddlesome attention which interferes with rest; upon the value of ventilation to afford the child an ample supply of natural oxygen. There seemed to be unanimity, also, upon the harmfulness of active medication, especialy with opium, nauseants, and antipyretics, also upon the necessity for care in the resort to stimulants. The hot poultice and cough mixture were inveighed against as relics of barbaric therapeutics.

Two of the speakers pithily expressed the object of the treatment of pneumonia of children to be "to treat the child, and not the disease"—to enhance the resisting power of the patient's organism by removing all debilitating influences and strengthening cardiac action.

Upon only one point was there a diversity of opinion. All the speakers referred with more or less approval to baths, the temperature of which ranged from 70° to 100° F.; and one speaker advised the wet sheet cooled by rubbing ice over it. This diversity in temperature and method of applying water is explained by the fact that all but one of the speakers advocated the remedial agent for the reduction of high temperature. The latter advised the mildest baths, the lowest temperature of which was 80° F., claiming that in pneumonia the resistance to temperature reduction is so diminished that great caution should be exercised with regard to low bath temperatures. He insisted that it is the chief object of the baths to neutralize the effect of toxemia and stimulate the heart. Another speaker, on the other hand, warned against all baths, warm or cold, when the heart action is feeble, stating that he regarded 70° F. as a warm bath. This may account for his warning; for a bath of 70° F. for five to ten minutes, whether it be regarded as warm or cold, would be a heroic measure for a child suffering from pneumonia.

The happy mean advocated by a majority of the speakers would seem the safest and wisest proceeding.

The section is to be congratulated upon the felicitous presentation of every aspect of this important subject.—*Editorial in the Record.*

